# Clinical pharmacy services are performed at several emergency wards in Denmark

**DOI:** 10.1186/1757-7241-20-S2-P9

**Published:** 2012-04-16

**Authors:** Susie Vand, Lene Juel Kjeldsen, Trine Kart

**Affiliations:** 1 The Hospital Pharmacy Århus, Horsens Department, Horsens, Denmark; 2 SAFE, Amgros I/S, Copenhagen Ø, Denmark

## Background

When establishing new emergency wards, the clinical pharmacy profession has been included at some hospitals with the aim of assisting in optimizing medication treatment for admitted patients. However, it is unknown to what extent clinical pharmacy is performed and at which hospitals. To develop a national strategy and improve communication about the effects of clinical pharmacy, the Danish hospital pharmacies have requested a description of existing clinical pharmacy activities at emergency wards. Hence, the aim of the study was to describe clinical pharmacy activities at emergency wards in Denmark.

## Methods

To ensure a 100% response rate, a study group containing one member from each region in Denmark was established. The 10 hospital pharmacies in Denmark were contacted with questions regarding type and extent of clinical pharmacy performed by clinical pharmacists and pharmaconomists at emergency wards in Denmark as well as how the services were funded. Data were collected in December 2010.

## Results

Logistic services were provided by all 10 hospital pharmacies to 20 emergency wards. These services included ordering of medication according to the wards’ standard assortment and local medication recommendations, and were delivered by pharmaconomists. Clinical pharmacists provided services to 15 emergency wards by performing e.g.: medication reviews (14), addressing medication-related questions (13), and teaching physicians and nurses (5). Other tasks included monitoring electronic medication charts, reporting of adverse events, performing internal audits, acquiring patient medication histories, conducting medication reconciliation, attending conferences and performing patient interviews. At 11 wards, established contracts had been made regarding the clinical pharmacist services, while project activities were delivered to 4 wards. Funding came from the emergency wards, the hospital pharmacy, the hospital management or external funding.

## Conclusion

The study showed that all hospital pharmacies delivered clinical pharmacy services to 20 emergency wards although the services provided varied in content and extent. Logistic clinical pharmacy services provided by pharmaconomists were performed at each of the 20 emergency wards, while a range of variable cognitive clinical pharmacy services were delivered by clinical pharmacists. The study may be used to develop a common concept for Danish clinical pharmacy at emergency wards.

